# Genotypic analysis of RTS,S/AS01_E_ malaria vaccine efficacy against parasite infection as a function of dosage regimen and baseline malaria infection status in children aged 5–17 months in Ghana and Kenya: a longitudinal phase 2b randomised controlled trial

**DOI:** 10.1016/S1473-3099(24)00179-8

**Published:** 2024-09

**Authors:** Michal Juraska, Angela M Early, Li Li, Stephen F Schaffner, Marc Lievens, Akanksha Khorgade, Brian Simpkins, Nima S Hejazi, David Benkeser, Qi Wang, Laina D Mercer, Samuel Adjei, Tsiri Agbenyega, Scott Anderson, Daniel Ansong, Dennis K Bii, Patrick B Y Buabeng, Sean English, Nicholas Fitzgerald, Jonna Grimsby, Simon K Kariuki, Kephas Otieno, François Roman, Aaron M Samuels, Nelli Westercamp, Christian F Ockenhouse, Opokua Ofori-Anyinam, Cynthia K Lee, Bronwyn L MacInnis, Dyann F Wirth, Peter B Gilbert, Daniel E Neafsey

**Affiliations:** a Fred Hutchinson Cancer Center, Vaccine and Infectious Disease Division, Seattle, WA, USA; b Broad Institute, Infectious Disease and Microbiome Program, Cambridge, MA, USA; c GSK, Wavre, Belgium; d Harvard T.H. Chan School of Public Health, Department of Biostatistics, Boston, MA, USA; e Emory University Rollins School of Public Health, Department of Biostatistics and Bioinformatic, Atlanta, GA, USA; f Department of Statistics, University of Washington, Seattle, WA, USA; g PATH, Seattle, WA, USA; h Kwame Nkrumah University of Science and Technology/Agogo Presbyterian Hospital, Agogo, Asante Akyem, Ghana; i Centre for Global Health Research, Kenya Medical Research Institute, Kisumu, Kenya; j Malaria Branch, Division of Parasitic Diseases and Malaria, Center for Global Health, Centers for Disease Control and Prevention, Kisumu, Kenya; k Malaria Branch, Division of Parasitic Diseases and Malaria, Center for Global Health, Centers for Disease Control and Prevention, Atlanta, GA, USA; l Harvard T.H. Chan School of Public Health, Department of Immunology and Infectious Diseases, Boston, MA, USA; m Department of Biostatistics, University of Washington, Hans Rosling Center for Population Health, Seattle, WA, USA

## Abstract

**Background:**

The first licensed malaria vaccine, RTS,S/AS01_E_, confers moderate protection against symptomatic disease. Because many malaria infections are asymptomatic, we conducted a large-scale longitudinal parasite genotyping study of samples from a clinical trial exploring how vaccine dosing regimen affects vaccine efficacy.

**Methods:**

Between Sept 28, 2017, and Sept 25, 2018, 1500 children aged 5–17 months were randomly assigned (1:1:1:1:1) to receive four different RTS,S/AS01_E_ regimens or a rabies control vaccine in a phase 2b open-label clinical trial in Ghana and Kenya. Participants in the four RTS,S groups received two full doses at month 0 and month 1 and either full doses at month 2 and month 20 (group R012-20); full doses at month 2, month 14, month 26, and month 38 (group R012-14); fractional doses at month 2, month 14, month 26, and month 38 (group Fx012-14; early fourth dose); or fractional doses at month 7, month 20, and month 32 (group Fx017-20; delayed third dose). We evaluated the time to the first new genotypically detected infection and the total number of new infections during two follow-up periods (12 months and 20 months) in more than 36 000 dried blood spot specimens from 1500 participants. To study vaccine effects on time to the first new infection, we defined vaccine efficacy as one minus the hazard ratio (HR; RTS,S *vs* control) of the first new infection. We performed a post-hoc analysis of vaccine efficacy based on malaria infection status at first vaccination and force of infection by month 2. This trial (MAL-095) is registered with ClinicalTrials.gov, NCT03281291.

**Findings:**

We observed significant and similar vaccine efficacy (25–43%; 95% CI union 9–53) against first new infection for all four RTS,S/AS01_E_ regimens across both follow-up periods (12 months and 20 months). Each RTS,S/AS01_E_ regimen significantly reduced the mean number of new infections in the 20-month follow-up period by 1·1–1·6 infections (95% CI union 0·6–2·1). Vaccine efficacy against first new infection was significantly higher in participants who were infected with malaria (68%; 95% CI 50–80) than in those who were uninfected (37%; 23–48) at the first vaccination (p=0·0053).

**Interpretation:**

All tested dosing regimens blocked some infections to a similar degree. Improved vaccine efficacy in participants infected during vaccination could suggest new strategies for highly efficacious malaria vaccine development and implementation.

**Funding:**

GlaxoSmithKline Biologicals SA, PATH, Bill & Melinda Gates Foundation, and the German Federal Ministry of Education and Research.

## Introduction

Malaria infection by the *Plasmodium falciparum* parasite causes over 230 million cases and 600 000 deaths per year, and progress in reducing morbidity and mortality through vector control and drug treatment has stalled.^1^ RTS,S/AS01_E_ (GSK, Wavre, Belgium)—referred to throughout as RTS,S—is the first vaccine recommended for *P falciparum* malaria by WHO and it provides moderate protective efficacy against clinical malaria. Improving protective efficacy is a major goal of ongoing work, including testing alternative dosing schedules and gaining a greater understanding of the mechanism of protection.

Most malaria vaccine trials evaluate vaccine efficacy using clinical disease as an outcome, but enhanced understanding of the mechanism and magnitude of protection could be gained from the molecular detection of new infections, given that a large proportion of malaria infections are asymptomatic.^2^ In this trial, we report on the MAL-095 study, a genotyping investigation of infection outcomes using samples from the MAL-094 phase 2b open-label randomised controlled trial of RTS,S. The MAL-094 trial enrolled children aged 5–17 months in Ghana and Kenya and used clinical disease outcomes to investigate the effect of dosing regimen on vaccine efficacy, ultimately finding no significant differences in vaccine efficacy against clinical disease between a delayed third dose regimen (R017), a fractional third dose regimen (Fx012), and the standard full-dose regimen (R012).^3^

**Research in context**

**Evidence before this study**

So far, it has been challenging to develop a long-lasting and highly effective vaccine against malaria caused by *Plasmodium falciparum*. Protective vaccine efficacy against malaria and other infectious diseases may be measured in different ways (outcomes). We searched PubMed for articles published before Dec 3, 2023, using the search terms “malaria”, “vaccine”, “infection”, and “endpoint”. Our search yielded 30 results. Most clinical trials of RTS,S/AS01_E_, the first licensed malaria vaccine, as well as other vaccine candidates, have used clinical (symptomatic) disease as a measure of protective efficacy in randomised controlled field trials. Some studies, including controlled human malaria infection studies, have used PCR to detect first infections after vaccination as an outcome.

**Added value of this study**

To our knowledge, this study is the first to use DNA sequencing of highly variable parasite genes on a large longitudinal collection of blood samples from clinical vaccine trial participants to fully profile infection status and dynamics before and after vaccination. Because many malaria infections are asymptomatic, they may not manifest as clinical disease. And in high-transmission settings, participants could be infected with multiple distinct parasite strains that are not resolvable via PCR. This study shows that different dosage regimens of RTS,S/AS01_E_ do not significantly change vaccine efficacy against infection. We further unexpectedly report a positive association between infection status during the first RTS,S/AS01_E_ vaccination and vaccine efficacy against infection.

**Implications of all the available evidence**

This study shows the value of genotyping for understanding malaria vaccine protection against an infection outcome and motivates new studies of RTSS/AS01_E_ and other malaria vaccines to further evaluate the relationship between malaria infection risk, malaria infection status at vaccination, and protective vaccine efficacy.

To explore protection conferred by each regimen in that study using a molecular infection outcome, we genotyped more than 36 000 blood samples taken both at symptomatic clinic visits and at monthly cross-sectional timepoints. We used a genotyping assay that detects infections at a sub-microscopic scale and distinguishes newly incident superinfections from persistent asymptomatic infections, yielding the capacity to measure both the time to first new infection and the cumulative number of new parasite infections after vaccination. We additionally assessed vaccine efficacy according to genotype of the infecting parasites given the previous observation of allele-specific vaccine efficacy in the phase 3 RTS,S trial.^4^ Because our genotyping assay detects newly incident superinfections in individuals with pre-existing infections, we performed a post-hoc analysis of vaccine efficacy on the basis of infection status at first vaccination to test the hypothesis cited in other studies published in 2021 that an erythrocytic malaria infection during vaccination impairs development of a protective immune response.^5^, ^6^

## Methods

### Study design and participants

As described in the primary analysis of the parent study (MAL-094; NCT03276962) evaluating protection against clinical disease,^3^ 1500 participants aged 5–17 months were enrolled across the study sites in Agogo, Ghana, and Siaya, Kenya, in this phase 2b randomised controlled trial.^3^ All participants in the exposed set of the parent study were enrolled in this study. No additional inclusion or exclusion criteria were used. Before the start of the parent study, both sites were assessed as having perennial, moderate-to-high *P falciparum* transmission with Kenya having a prevalence approximately double that of Ghana (39% *vs* 17%, as estimated by microscopy).^3^ The trial protocol was approved by all relevant ethical review boards at the study sites and investigator institutions.

Signed or witnessed thumbprint informed consent was obtained from the children's parents or guardians before participation. Sex and gender data were reported by parents or guardians.

### Randomisation and masking

**Figure 1:**
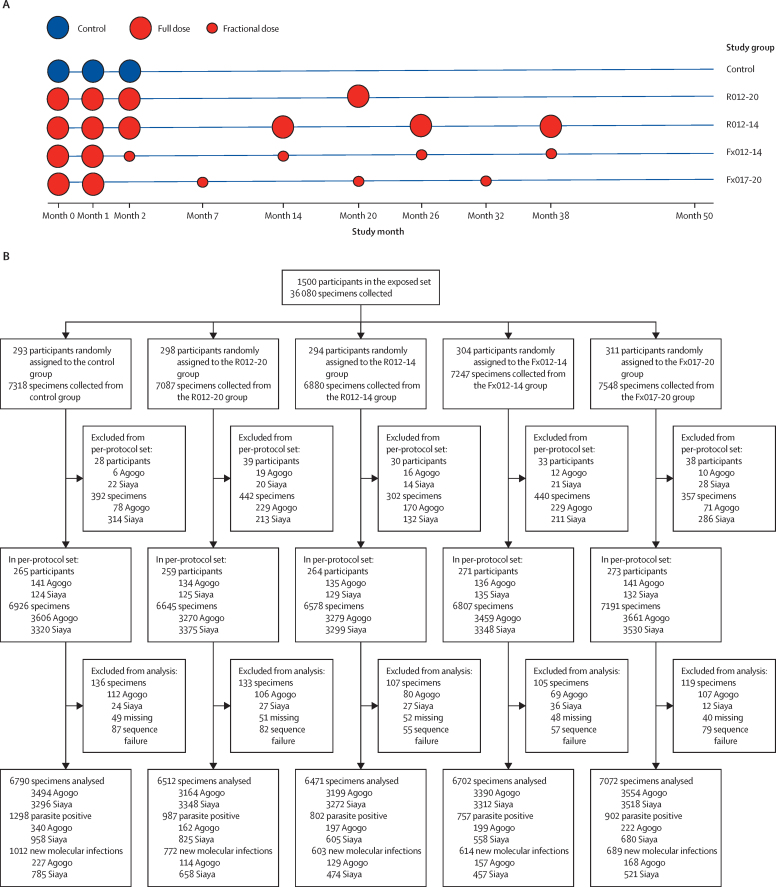
Vaccination and dosage schedule for the full parent trial (A) and specimen collection and genotype data generation by study group in the per-protocol set through to month 20 (B) Samples were collected passively during febrile clinic visits and cross-sectionally at monthly intervals through to study month 20 and at 3-monthly intervals between month 20 and month 32.

Participants were randomly assigned into one of five vaccination groups (1:1:1:1:1) for the purposes of the parent study.^3^ The control rabies vaccination group was vaccinated at month 0, month 1, and month 2. Participants in the four RTS,S groups received two full doses at month 0 and month 1 and either full doses at month 2 and month 20 (group R012-20); full doses at month 2, month 14, month 26, and month 38 (group R012-14); fractional doses at month 2, month 14, month 26, and month 38 (group Fx012-14; early fourth dose); or fractional doses at month 7, month 20, and month 32 (group Fx017-20; delayed third dose; figure 1, appendix p 20). The participants were identified by identification numbers.

### Procedure

Participant samples were collected as dried blood spots (DBS) on Whatman FTA sample cards at the baseline enrolment visit (study month 0), cross-sectionally at monthly intervals until study month 20, cross-sectionally at three-month intervals between study month 20 and month 32, and during febrile clinic visits. Blood smears were collected for microscopy-based detection of infection. For individuals who were asymptomatic, blood smears were evaluated at a later date and did not trigger treatment to clear infection. Participants meeting the primary or secondary case definitions of clinical malaria were treated according to the national guidelines of each country, with the primary case definition being more than 5000 asexual parasites per μL and fever (axillary temperature ≥37·5°C), and the secondary case definition being any parasitaemia (ie, more than zero parasites per μL) and fever or history of fever within 24 h of presentation.

We analysed all DBS samples by extracting DNA and performing Illumina-based amplicon sequencing of the circumsporozoite protein C-terminus coding region and a comparably polymorphic coding region for the antigen serine repeat antigen 2 (SERA2). We defined distinct haplotypes as the combined genotype of all nucleotide variants in each amplicon sequence. Complexity of infection (COI) was defined as the maximum number of distinct haplotypes detected in a sample at either amplicon. We declared a new parasite infection on a specific sampling date if at least one haplotype was observed for either amplicon that had not been previously detected in the preceding three sample timepoints from that individual. Haplotype diversity was high at both study sites for both the circumsporozoite and SERA2 amplicons, making it extremely unlikely that two distinct infections in the same host would harbour the same circumsporozoite and SERA2 haplotypes (appendix p 21). A full description of molecular methods, data filtration, and sequence analysis is in the appendix (pp 8–14). All sequencing data were submitted to the NCBI Sequence Read Archive (BioProject PRJNA983279).

### Outcomes

The primary outcomes measured in this study were the time to the first new malaria infection and the number of new malaria infections acquired over time, as measured by genotyping (ie, genotypic infection). The secondary outcome measured was the parasite genotype. Post-hoc analyses were conducted to assess the primary outcomes considering baseline infection status and the cumulative number of new genotypic infections (force of infection [FOI]) detected after the first vaccination visit and by the visit conducted 2 months after the first vaccination (ie, the month 2 visit).

### Statistical analysis

Sample size was determined by power considerations for the clinical outcomes investigated in the parent study of this trial. All analyses planned before the execution of this study are described in the statistical analysis plan included in the appendix (p 62). For this study, we first assessed vaccine efficacy of each RTS,S regimen versus the rabies control vaccine and relative vaccine efficacy comparing the RTS,S regimens head-to-head to prevent the first new genotypic infection and to reduce the number of new genotypic infections. These analyses were performed in parallel for the exposed set of participants who received at least the first vaccine dose and the per-protocol set of participants who received the first three doses of the vaccine per protocol and were in primary follow-up at 14 days after the third dose (ie, these participants had the potential for future follow-up visits; figure 1B; appendix p 20). We analysed the follow-up period from the first dose to the visit at month 20 in the exposed set, and from 14 days after the third dose to a visit scheduled 12 months after the third dose in the per-protocol set. To explore vaccine efficacy over a longer interval, we also analysed a follow-up period from the first dose to the visit at month 32 in the exposed set, and from 14 days to a visit scheduled 24 months after the third dose in the per-protocol set.

To study vaccine effects on time to the first new infection, we defined vaccine efficacy as one minus the hazard ratio (HR; RTS,S *vs* control) of the first new infection estimated using the Cox proportional hazards model with 95% Wald CIs and two-sided Wald tests of zero vaccine efficacy. For RTS,S head-to-head comparisons, relative vaccine efficacy was defined analogously by replacing the control with an active comparator regimen. Furthermore, we estimated instantaneous vaccine efficacy over time with 95% pointwise and simultaneous CIs using non-parametric kernel-smoothing^7^ and tested for variation in vaccine efficacy across time.^8^ Cumulative incidence of the first new infection was estimated using the transformed Nelson-Aalen estimator for the cumulative hazard function.

We measured vaccine effects on the number of new infections by the additive difference (RTS,S *vs* comparator) in the mean number of new infections. The infection count was defined as unobserved if the number of missed visits or samples exceeded a specified threshold (appendix p 90). We assessed the mean difference by targeted maximum likelihood estimation (TMLE),^9^ accounting for unobserved infection counts. Additionally, we used TMLE to estimate reverse cumulative distribution functions of the number of new infections in each study group. Besides overall vaccine efficacy, in the per-protocol set we assessed whether and how vaccine efficacy against the first new infection depended on genotypic characteristics of infecting parasites using augmented inverse probability weighting methods^10^, ^11^ and their complete-case analogues (appendix pp 15, 93).^12^

Furthermore, in a post-hoc analysis assessed in the per-protocol set, we evaluated whether baseline parasite positivity or infection risk, or both, modified the effect of RTS,S on the time to the first new genotypic infection and the time to the first new clinical malaria episode. Covariate-adjusted Cox proportional hazards models with separate baseline hazards for each study site, employing 95% Wald CIs, Wald interaction tests, and Nelson−Aalen-based cumulative incidence curves were used. We performed a sensitivity matching Cox analysis with stratified sampling, wherein participants who were parasite negative at first vaccination were randomly sampled from the same randomisation group and study site by matching participants who were parasite positive at first vaccination on the date of the third vaccination to address potential confounding by low-transmission versus high-transmission season. Additionally, a sensitivity analysis with E-values quantified the robustness of evidence for baseline parasite positivity and infection risk causally modifying vaccine efficacy (appendix p 16).

All analyses were performed on pooled data from both study sites and separately within each site. Tests for vaccine efficacy departing from zero were adjusted for multiplicity separately within each analysis cohort, study site-pooled versus study site-specific analysis, and each of the three sets of treatment comparison types defined as follows: comparisons versus the control regimen other than the primary comparisons of each of Fx012-14 and Fx017-20 versus control, comparisons versus the standard R012-20 RTS,S regimen, and head-to-head comparisons of novel RTS,S regimens (appendix p 84). For each multiplicity set, p value adjustments were implemented to control the family-wise error rate (FWER; Holm−Bonferroni^13^) and the false discovery rate (FDR; Q-values; Benjamini−Hochberg^14^). We defined FWER statistical significance as an FWER-adjusted p value of 0·05 or less and FDR statistical significance as a Q-value of 0·2 or less together with an unadjusted p value of 0·05 or less. All p values are two sided except p values testing differential vaccine efficacy by 3D7 Hamming distances and by COI, which are double one-sided. R version 4.2.3 was used for the analyses.

### Role of the funding source

Two of the funders (GlaxoSmithKline Biologicals SA and PATH) participated in the study design, data collection, data analysis, data interpretation, and writing of the report.

## Results

This study included all participants from the exposed set of the parent study; no additional criteria were imposed.^3^ The exposed set comprised 1500 children, with 750 (50·0%) in Agogo and 750 (50·0%) in Siaya. Baseline characteristics are provided in the table.^3^ In the exposed set, 36 080 DBS samples were collected between the first dose and the visit at month 20. Of these specimens, 35 456 (98·3%) had genotyping completed; samples classified as missing or as sequencing failures were excluded (appendix p 20). Molecular detection classified 5078 (14·3%) of the 35 456 samples as parasite positive, whereas microscopy had identified 4115 (11·6%) of the 35 456 samples as parasite positive (concordance 0·74 by Cohen's kappa; appendix p 22). Of the parasite-positive samples identified by molecular detection, 3937 (77·5%) were associated with a new infection. The per-protocol set comprised 1332 children, with 687 (51·6%) in Agogo and 645 (48·4%) in Siaya. In the per-protocol set, 34 147 specimens were collected during the follow-up period (14 days to 12 months after the third dose). Of these 34 147 samples, 33 547 (98·2%) had genotyping completed and excluded samples were classified as missing or a sequencing failure (figure 1B). Of these 33 547 samples, 4746 (14·1%) were confirmed parasite positive by molecular detection, among which 3690 (77·7%) were associated with a new infection. In the exposed set and the per-protocol set, 1030 (68·7%) and 763 (57·3%) participants, respectively, had the first new genotypic infection during the respective follow-up period (ie, the first dose to the visit at month 20 in the exposed set, and from 14 days after the third dose to a visit scheduled 12 months after the third dose in the per-protocol set). The median time from the first dose to the first new infection in the exposed set was 39·7 weeks (IQR 14·0–85·0). The median time from the third dose to the first new infection in the per-protocol set was 37·0 weeks (16·8–not reached). The mean number of new genotypic infections per individual was 2·9 (SD 3·5) in the exposed set and 1·5 (2·0) in the per-protocol set.

**Table:** Baseline characteristics, combined and by country, for the exposed set and the per-protocol set

		**Control**	**R012-20**	**R012-14**	**Fx012-14**	**Fx017-20**
**Exposed set**						
Participant count		293	298	294	304	311
	Ghana	147 (50%)	153 (51%)	151 (51%)	148 (49%)	151 (49%)
	Kenya	146 (50%)	145 (49%)	143 (49%)	156 (51%)	160 (51%)
Male participants		141 (48%)	179 (60%)	140 (48%)	132 (43%)	148 (48%)
	Ghana	65/141 (46%)	93/179 (52%)	68/140 (49%)	64/132 (48%)	76/148 (51%)
	Kenya	76/141 (54%)	86/179 (48%)	72/140 (51%)	68/132 (52%)	72/148 (49%)
Female participants		152 (52%)	119 (40%)	154 (52%)	172 (57%)	163 (52%)
	Ghana	82/152 (54%)	60/119 (50%)	83/154 (54%)	84/172 (49%)	75/163 (46%)
	Kenya	70/152 (46%)	59/119 (50%)	71/154 (46%)	88/172 (51%)	88/163 (54%)
Age at first vaccination, months		10·5 (3·9)	10·2 (3·9)	10·3 (3·8)	10·5 (4·0)	10·2 (3·8)
	Ghana	10·4 (4·0)	9·7 (3·9)	10·4 (4·0)	10·1 (4·0)	10·2 (4·1)
	Kenya	10·7 (3·8)	10·7 (3·7)	10·1 (3·6)	10·9 (4·0)	10·2 (3·6)
BMI, kg/m^2^		16·5 (1·4)	16·9 (1·6)	16·7 (1·6)	16·9 (1·6)	16·8 (1·8)
	Ghana	16·1 (1·2)	16·4 (1·4)	16·2 (1·4)	16·4 (1·4)	16·2 (1·6)
	Kenya	17·0 (1·5)	17·4 (1·6)	17·2 (1·7)	17·3 (1·7)	17·3 (1·7)
Haemoglobin, g/dL		10·3 (1·1)	10·1 (1·1)	10·3 (1·1)	10·4 (1·1)	10·3 (1·1)
	Ghana	10·7 (1·0)	10·5 (1·1)	10·7 (1·0)	10·7 (1·1)	10·6 (1·0)
	Kenya	9·9 (1·2)	9·7 (1·1)	9·9 (1·1)	10·1 (1·1)	10·0 (1·0)
Height, cm		71·2 (5·3)	70·7 (5·3)	71·0 (5·3)	70·8 (5·2)	70·7 (5·1)
	Ghana	71·8 (5·4)	70·4 (5·6)	71·4 (5·3)	71·0 (5·6)	71·4 (5·6)
	Kenya	70·7 (5·1)	71 (5·0)	70·7 (5·2)	70·6 (4·7)	70·1 (4·5)
Weight, kg		8·4 (1·3)	8·5 (1·3)	8·5 (1·5)	8·5 (1·4)	8·4 (1·4)
	Ghana	8·3 (1·4)	8·2 (1·4)	8·3 (1·4)	8·3 (1·4)	8·3 (1·6)
	Kenya	8·5 (1·3)	8·8 (1·3)	8·7 (1·6)	8·6 (1·3)	8·5 (1·2)
**Per-protocol set**						
Participant count		265	259	264	271	273
	Ghana	141 (53%)	134 (52%)	135 (51%)	136 (50%)	141 (52%)
	Kenya	124 (47%)	125 (48%)	129 (49%)	135 (50%)	132 (48%)
Male participants		128 (48%)	151 (58%)	128 (48%)	115 (42%)	131 (48%)
	Ghana	60/128 (47%)	77/151 (51%)	64/128 (50%)	58/115 (50%)	68/131 (52%)
	Kenya	68/128 (53%)	74/151 (49%)	64/128 (50%)	57/115 (50%)	63/131 (48%)
Female participants		137 (52%)	108 (42%)	136 (52%)	156 (58%)	142 (52%)
	Ghana	81/137 (59%)	57/108 (53%)	71/136 (52%)	78/156 (50%)	73/142 (51%)
	Kenya	56/137 (41%)	51/108 (47%)	65/136 (48%)	78/156 (50%)	69/142 (49%)
Age at first vaccination, months		10·5 (3·8)	10·3 (3·9)	10·2 (3·8)	10·3 (3·9)	10·1 (3·9)
	Ghana	10·3 (3·9)	9·7 (4·0)	10·3 (4·0)	9·8 (3·9)	10·2 (4·1)
	Kenya	10·8 (3·6)	10·9 (3·7)	10·1 (3·5)	10·8 (3·9)	10·1 (3·5)
BMI, kg/m^2^		16·5 (1·4)	16·8 (1·5)	16·8 (1·6)	16·8 (1·6)	16·7 (1·7)
	Ghana	16·1 (1·2)	16·4 (1·3)	16·3 (1·4)	16·4 (1·4)	16·2 (1·6)
	Kenya	16·9 (1·5)	17·2 (1·6)	17·3 (1·7)	17·2 (1·6)	17·3 (1·7)
Haemoglobin, g/dL		10·3 (1·1)	10·1 (1·1)	10·3 (1·1)	10·4 (1·1)	10·3 (1·1)
	Ghana	10·7 (1·0)	10·5 (1·0)	10·7 (0·9)	10·7 (1·0)	10·6 (1·0)
	Kenya	9·9 (1·1)	9·7 (1·1)	9·9 (1·1)	10·2 (1·1)	10·0 (1·1)
Height, cm		71·4 (5·1)	70·8 (5·4)	70·9 (5·1)	70·6 (5·1)	70·6 (5·1)
	Ghana	71·8 (5·4)	70·4 (5·7)	71·3 (5·2)	70·7 (5·5)	71·3 (5·6)
	Kenya	70·9 (4·8)	71·2 (5·0)	70·6 (5·0)	70·5 (4·7)	69·9 (4·5)
Weight, kg		8·4 (1·3)	8·4 (1·4)	8·5 (1·5)	8·4 (1·3)	8·3 (1·4)
	Ghana	8·3 (1·4)	8·2 (1·4)	8·3 (1·4)	8·2 (1·4)	8·3 (1·6)
	Kenya	8·5 (1·3)	8·7 (1·3)	8·6 (1·6)	8·6 (1·2)	8·4 (1·2)

Data are n (%), n/N (%), or mean (SD).

**Figure 2:**
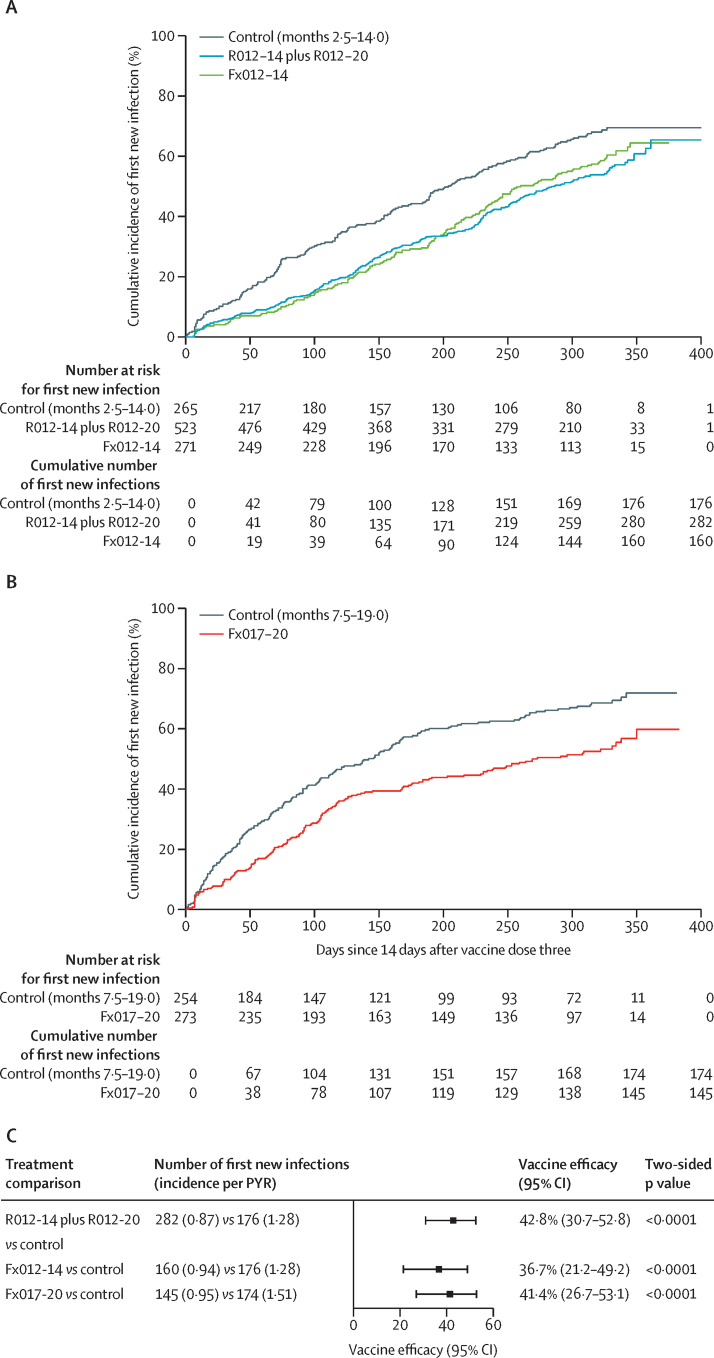
Cumulative incidence (A, B) and vaccine efficacy (C) in the per-protocol set against the first new genotypic infection between months 2·5–14·0 for R012-14 plus R012-20 and Fx012-14 *vs* the control regimen and between months 7·5–19·0 for Fx017-20 *vs* the control regimen PYR=person-year at risk.

The vaccine efficacy of each RTS,S regimen versus the control regimen was 25–31% (95% CI union 9–43) in the exposed set and 37–43% (21–53) in the per-protocol set, each significantly different from zero (all p<0·0033 in the exposed set and all p<0·0001 in the per-protocol set; figure 2; appendix p 23). No significant differences in the hazard rate of the first new infection were found in head-to-head comparisons of RTS,S regimens (all p>0·32; appendix p 24). Instantaneous vaccine efficacy over time suggests that the full dose at month 2 might have provided more sustained protection than a fractional dose at month 2, because the vaccine efficacy of Fx012-14 waned to zero by 7 months after the third dose (appendix p 25).

**Figure 3:**
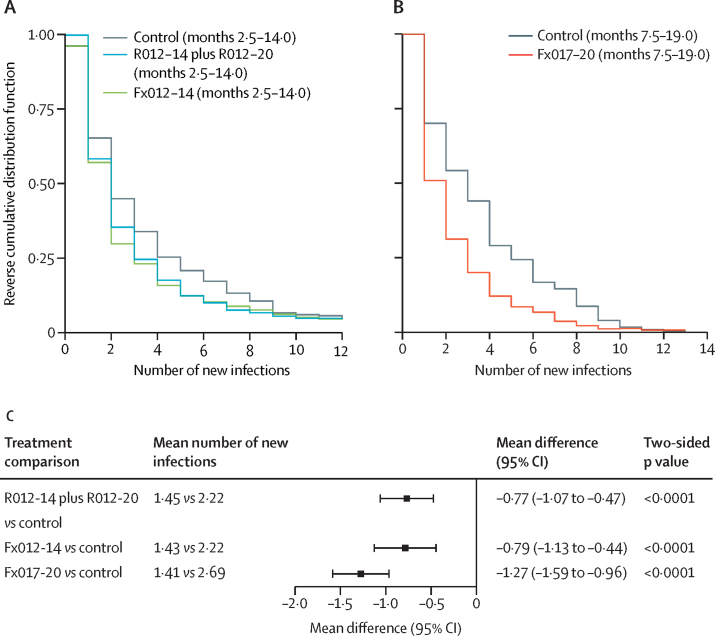
Reverse cumulative distribution function (A, B) and vaccine effect (C) on the mean number of new genotypic infections in the per-protocol set between months 2·5–14·0 for R012-14 plus R012-20 and Fx012-14 *vs* the control regimen and between months 7·5–19·0 for Fx017-20 *vs* the control regimen

The mean number of new infections in recipients of the RTS,S vaccine was significantly lower than that in recipients of the control vaccine in both the exposed set and the per-protocol set (all p<0·0001; figure 3; appendix p 26). In the exposed set, the mean new infection count during 20 months ranged between 2·6–3·0 among recipients of the RTS,S vaccine and was 4·1 among recipients of the control vaccine, with the mean difference ranging –1·6 to –1·1 (95% CI union, –2·1 to –0·6). In the per-protocol set, the mean new infection count between 14 days and 12 months after the third dose ranged between 1·4–1·5 among recipients of the RTS,S vaccine and was 2·2 and 2·7 among recipients of the control vaccine between months 2·5–14·0 or 7·5–19·0, respectively, with the mean difference ranging from –1·3 to –0·8 (–1·6 to –0·4).

**Figure 4:**
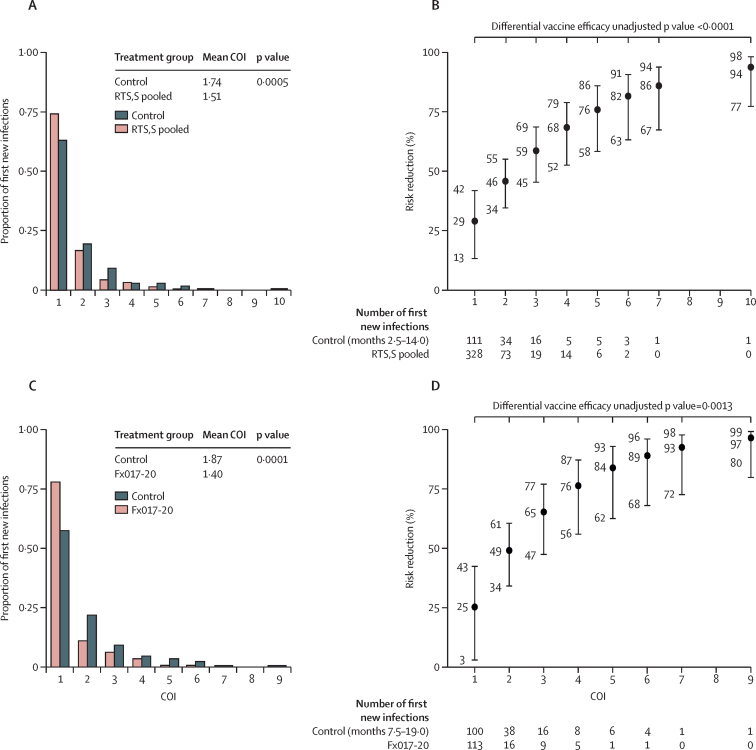
Complexity of first new genotypic infection between months 2·5–14·0 for the pooled R012-14, R012-20, and Fx012-14 *vs* the control regimen and between months 7·5–19·0 for Fx017-20 *vs* the control regimen in the per-protocol set: frequencies (A, C) and risk reduction (1–HR; B, D) against first new genotypic infection with a given complexity COI=complexity of infection. HR=hazard ratio.

RTS,S regimens diminished the COI pertaining to the first new infection compared with the control regimen in the per-protocol set (figure 4A, 4C). Moreover, RTS,S regimens showed a significantly greater reduction in the risk of more highly polyclonal first new infections (figure 4B, 4D). The estimated risk reduction of pooled R012-14, R012-20, and Fx012-14 versus control was 29% (95% CI 13–42) against single-haplotype first new infections and 76% (58–86) against first new infections with five haplotypes (p<0·0001 for increasing risk reduction with COI).

In the exploratory outcome analysis assessed in the per-protocol set, the genotypic sieve analysis was underpowered given the low 3D7 parasite haplotype prevalence and small outcome counts. No evidence was found for differential vaccine efficacy against the first new infection with a perfect amino acid residue match versus mismatch to the 3D7 vaccine strain in the circumsporozoite C-terminus full amplicon or haplotypic regions (appendix p 27). There was a non-significant vaccine efficacy decline with an increasing degree of residue mismatch to 3D7 in the circumsporozoite C-terminus (appendix p 28). Scanning individual polymorphic amino acid positions, we found hypothesis-generating signals of differential vaccine efficacy of Fx012-14 against first new infection strains with a match versus mismatch to a 3D7 residue at circumsporozoite C-terminus amino acid positions 322, 324, and 327 in Th2R (appendix pp 29–32).

In the per-protocol set, 154 (11·6%) of 1328 participants (51 [7·4%] of 686 in Agogo and 103 [16·0%] of 642 in Siaya) were parasite positive at first vaccination (referred to as baseline) by microscopic or genotypic assay, or both. The incidence rate of the first new infection in the control group was higher in participants who were parasite positive at baseline (3·0 per person-year at risk [PYR]) than in participants who were parasite negative at baseline (1·2 per PYR), suggesting a correlation between baseline positivity and infection risk.

Therefore, we also analysed the cumulative number of new genotypic infections detected after the first vaccination visit and by the month 2 visit (molecular force of infection by month 2, referred to as M2-FOI). This covariate is an aggregate proxy of individual-level infection risk due to many factors including seasonal transmission effects, local geography, susceptibility to mosquito bites, and malaria prevention use. M2-FOI could potentially confound the vaccine efficacy-modifying effect of baseline positivity, because M2-FOI was correlated with baseline positivity and the calendar date of the first vaccination (appendix pp 33–34). We also accounted for such potential confounding by adjusting for the indicator of the onset of antimalarial drug treatment between the first vaccination visit and the month 2 visit (referred to as M2-mal-tx), which correlated with baseline positivity (appendix p 35). Additional vaccine efficacy-modification analyses were conducted adjusting for M2-FOI and M2-mal-tx, an adjustment with minimal risk of post-randomisation selection bias because vaccination had no discernible effect on M2-FOI or M2-mal-tx (appendix pp 36–37).

**Figure 5:**
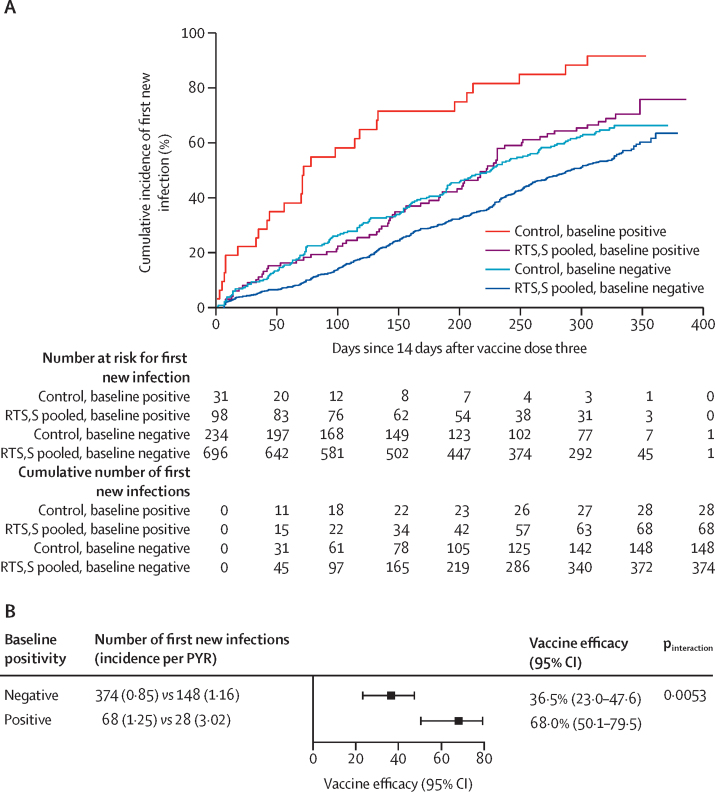
Cumulative incidence (A) and vaccine efficacy (B) against the first new genotypic infection between months 2·5–14·0 for the pooled R012-14, R012-20, and Fx012-14 *vs* the control regimen in the per-protocol set by baseline malaria infection status while adjusting for M2-FOI M2-FOI=number of new infections detected after the first vaccination visit and by the month 2 visit. PYR=person-year at risk.

Adjusting for M2-FOI, M2-mal-tx, sex, and age, vaccine efficacy of pooled R012-14, R012-20, and Fx012-14 versus control to prevent the first new genotypic infection in the per-protocol population was 37% (95% CI 23–48) among participants who were baseline negative and 68% (50–80) among participants who were baseline positive (interaction p_interaction_=0·0053; figure 5; appendix p 38). Vaccine efficacy modification by baseline positivity persisted when restricted to the early follow-up period between 14 days and 4·5 months after the third dose (p_interaction_=0·083; appendix p 39), a period exhibiting relatively little waning of vaccine efficacy. The evidence for baseline positivity as a modifier of vaccine efficacy was consistent across the two study sites, the individual RTS,S regimens with dosing at study months 0, 1, 2, and the full per-protocol versus sensitivity third vaccination matching Cox analysis (appendix pp 40–42).

**Figure 6:**
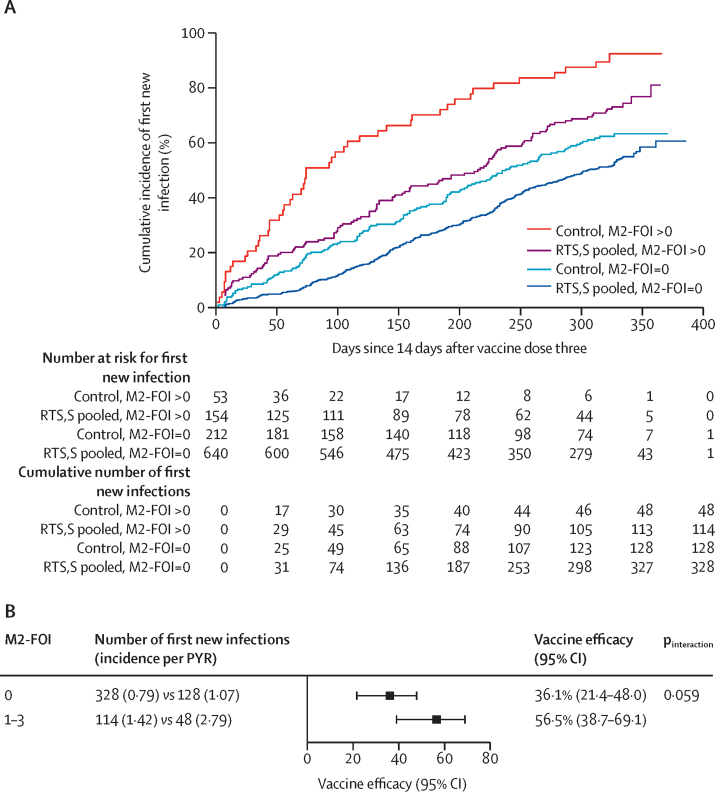
Cumulative incidence (A) and vaccine efficacy (B) against the first new genotypic infection between months 2·5–14·0 for the pooled R012-14, R012-20, and Fx012-14 *vs* the control regimen in the per-protocol set by M2-FOI equal to zero vs M2-FOI greater than zero M2-FOI=number of new infections detected after the first vaccination visit and by the month 2 visit. PYR=person-year at risk.

As an indicator of intercurrent malaria infection between dose one and dose three, M2-FOI has a distinct interpretation compared with baseline positivity, motivating an exploratory analysis of whether M2-FOI itself modifies vaccine efficacy. Adjusting for baseline positivity, M2-mal-tx, sex, and age, vaccine efficacy of the same pooled RTS,S groups versus control against the first new genotypic infection in the per-protocol set was 36% (95% CI 22–48) among participants with M2-FOI equal to zero and 57% (39–69) among participants with M2-FOI greater than zero (p_interaction_=0·059; figure 6; appendix p 38). Vaccine efficacy modification evidence from a series of Cox models involving both baseline positivity and M2-FOI, including model quality assessment, is summarised in the appendix (pp 16–19) for the genotypic infection outcome. A sensitivity analysis, reported in the appendix (pp 16–19), supported that the result of vaccine efficacy modification by baseline positivity was robust to unmeasured confounding.

We do not report the effect of either baseline positivity or M2-FOI on vaccine efficacy against the first new clinical malaria episode. Inference on these causal interaction effects is confounded by a differential propensity of first clinical episodes arising due to persistent asymptomatic infections acquired before the third vaccination, which are much more common in subgroups of participants who were parasite positive at baseline and those with M2-FOI greater than zero than in participants with baseline negative status and M2-FOI of zero, respectively (appendix p 43).

Participant numbers, genetic outcomes, and primary outcome vaccine efficacy estimates for the longer follow-up period (ie, from the first dose to the visit scheduled 32 months later in the exposed set and from 14 days after the third dose to the visit scheduled 24 months after the third dose in the per-protocol set) are reported in the appendix (pp 44–60) because they are highly similar to the results we have presented.

## Discussion

The use of genotypically determined infection outcomes in this study has shown unambiguously for the first time, to our knowledge, that RTS,S results in some or all of the vaccine efficacy observed through blocking of infections before they reach the blood stage, with the RTS,S groups showing a reduced number of new infections (figure 3) and a reduced risk of more highly polyclonal first infections (figure 4) compared with the control group. Although the previous analysis of parasite genotypic features we performed on specimens from the RTS,S phase 3 trial suggested this in the form of reduced COI,^4^ that study only analysed specimens from the first cases meeting the primary clinical case definition.

The genotypically determined infection outcomes yielded findings generally concordant with the previous analysis of clinical disease outcomes with regard to the effects of RTS,S vaccine dosage and regimen. Although all RTS,S dosage regimens offer significant vaccine efficacy, none of the regimens are superior for the follow-up period that we examined. The genotypic outcomes we examined suggest lower instantaneous vaccine efficacy in the Fx012-14 group several months after the third dose (appendix p 25) compared with the other RTS,S groups, suggesting that fractional dose regimens could offer slightly less protection than full dose regimens administered on the same schedule.

Additionally, this study shows several ways in which genotypically determined infection outcomes complement clinical disease or microscopy-based infection outcomes. We observed that vaccine efficacy against the first new genotypically detected infection was higher in participants who were parasite positive at baseline (ie, asymptomatically infected with malaria during their first vaccination) than in those who were parasite negative at baseline. Participants who had more infections between their first vaccination and month 2 visit (M2-FOI) did not exhibit abrogated vaccine efficacy and showed greater protection. This finding suggests that active infection, higher risk of infection, or both potentiate RTS,S vaccine efficacy. Because baseline infection status and M2-FOI are correlated and these features were not stratified in the study design, distinguishing their relative effects is difficult using the current data. Furthermore, we cannot presently distinguish whether variation in infection risk among participants is due to environmental, immunological, or other factors. However, active infections at the time of first vaccination could affect vaccine efficacy by the priming of circumsporozoite-specific T-helper cells provided by natural infection, resulting in enhanced production of protective antibodies, a more effective cellular immune response, or both during the liver stage. Similarly, the non-significant finding of greater vaccine efficacy observed in participants with M2-FOI greater than zero compared with those with M2-FOI equal to zero could be driven by repeated natural exposure to the circumsporozoite antigen from infectious mosquito bites as a form of heterologous prime-boost; however, the effect of an active infection on vaccine efficacy persists while controlling for M2-FOI.

The observation of increased RTS,S protection in participants who were infected at baseline or had M2-FOI greater than zero is unexpected. A large number of studies have documented immunosuppressive effects of acute or asymptomatic malaria infection in various human or rodent model contexts,^15^, ^16^, ^17^, ^18^, ^19^, ^20^ and these observations have led to the hypothesis that erythrocytic-stage malaria infection at the time of vaccination could compromise the development of an efficacious immune response, measured at the level of either clinical disease^5^ or molecularly detected infection.^6^ However, an analysis of RTS,S efficacy in the phase 3 clinical trial published in 2023 found that protection against clinical malaria was unaffected by infection status during vaccination.^21^ To our knowledge, our work is the first to show a positive association between erythrocytic infection present at the first vaccination and vaccine efficacy against infection, perhaps because no other study has used a similar genotypic analysis. Studies reporting discordant findings with regard to erythrocytic infections impairing immunity in controlled human malaria infection studies focusing on malaria-naive adults^6^ or mouse models^20^ could reflect fundamentally different mechanisms of pre-erythrocytic immunity development. Future studies will be required to understand this apparent discordance.

A limitation of our study is that we do not report on the effects of baseline positivity or M2-FOI on vaccine efficacy against the first new clinical malaria episode, because we cannot exclude the possibility that infections acquired before the third vaccination contribute to first new clinical malaria episodes, and such infections are more common in participants who are parasite-positive at baseline and those with M2-FOI greater than zero. Indeed, the genotypic profile of some baseline infections matches that of the first clinical episode in some participants (appendix p 43), and other studies have reported an increased risk of clinical disease in individuals with asymptomatic infections.^22^ Our results, however, and those of a serological study of a previous RTS,S phase 2b field study^23^ suggest that the protection afforded by RTS,S against clinical episodes largely derives from protection against infection, rather than attenuation of blood stage infection intensity. We therefore expect that in a study designed to properly measure the effects of parasite positivity at baseline or the M2-FOI, or both, on clinical disease, positive associations might be seen, but this requires confirmation.

There are several important consequences of the observation of greater RTS,S vaccine efficacy in association with baseline parasite positivity and infection risk, for both RTS,S and perhaps for the R21/Matrix-M vaccine recommended in 2023, which uses an identical circumsporozoite peptide subunit as RTS,S,^24^ and could show similar modulation of vaccine efficacy. Firstly, this finding indicates that future studies of the efficacy of RTS,S and other candidate malaria vaccines might need to take into account local transmission level or heterogeneity in infection risk among participants for randomisation, or both, because differential vaccine efficacy against infection or clinical disease as a function of baseline infection status, infection risk, or both could influence vaccine deployment strategy. Secondly, this finding motivates the inclusion of genotypic outcomes in future intervention studies to further assess the effects of baseline infection status and molecular FOI on protection against both infection and clinical disease, as well as immune assays to understand the mechanism of the protective effect.^25^ Thirdly, this finding could lead designers of future vaccine and monoclonal antibody field trials to re-evaluate the practice of diagnosing and clearing pre-existing malaria infections from participants during enrolment, which is a common approach^26^, ^27^, ^28^ that could limit the approved use of an intervention to uninfected recipients if it is later licensed.

Genotyping the monthly cross-sectional samples collected from all participants in this study has provided an unprecedented view of asymptomatic and polyclonal infection dynamics in a natural setting. The value of such data in malaria drug efficacy studies has been previously noted,^29^ and the portability of observations across intervention studies will be enhanced as the field develops common standards for genotyping data and analysis. The evaluation of future malaria interventions with genotyping data will enable direct measurement of their potential not only to mitigate clinical cases, but also to attain local disease elimination.

## Data sharing

Sequence data have been deposited with the Sequence Read Archive in association with BioProject PRJNA983279. The study protocol and statistical analysis plan will also be shared with publication. Reprint requests should be addressed to MJ and DEN.

## Declaration of interests

The findings and conclusions in this article are those of the authors and do not necessarily represent the views of the US Centers for Disease Control and Prevention or the US Department of Health and Human Services. LDM received grants from the Bill and Melinda Gates Foundation and the German Federal Ministry of Education and Research through the KfW Development Bank through her institution. CKL received a grant from the German Federal Ministry of Education and Research through the KfW Development Bank and a grant from the Bill and Melinda Gates Foundation. DFW acted as a principal investigator on the MAL-095 study funded by a PATH grant paid to Harvard University, which also supported DEN, AME, BLM, SFS, and AK. DFW is also Chair of the Malaria Policy Advisory Group that advises the WHO on all malaria policy. PBG discloses a PATH subaward from Harvard for statistical analysis contributing to salary support for PBG, MJ, and LL. AME, LL, AK, BS, NSH, DB, SaA, TA, ScA, DA, DKB, PBYB, SE, NF, JG, SKK, KO, AMS, NW, and CFO declare no conflict of interest. ML, FR, OO-A are employees of GSK. ML, FR, and OO-A own shares in GSK.
